# Co-Operative Colonies for Incurable Neurasthenics

**Published:** 1920-08-21

**Authors:** 


### CO-OPERATIVE COLONIES FOR INCURABLE NEURASTHENICS.

By A CORRESPONDENT.

It is, unfortunately, quite certain that a small proportion of the men rendered neurasthenic by the accidents of war—disease, fatigue, anxiety, injury, etc.—will never regain sufficient nervous stability, adaptability, and vigour for strenuous competitive life.

The central nervous system is essentially impressionable, and its impressions are bound, in some sense and to some degree, to be permanent either for good or evil. It is the growing evolving product of cumulative experiences, and each experience is permanently impressed on its molecular constitution, and included and compounded in its final functions. It may start existence as a *tabula rasa*, but the tabula soon becomes a palimpsest, and the earliest inscriptions are never quite obliterated. Every acute sensation, every keen emotion, every vivid thought leaves a permanent organic record. The brain of a man who has learned a new language has acquired new molecular arrangements; a man who has learned to play a piano has "blazed" new tracks in the tangled jungles of his nerve axons and dendrons; a man who faced and fought a great fear will have its mark upon his neurons as long as he lives. Indeed, on the persistence of the neural impressions of experience depend the whole neurological career and spiritual personality of a man. Both neurologically and spiritually speaking, a man's past, present, and future are inextricably interwoven. Even when past experiences seem to play no part in a man's present consciousness, they are subtly at work, for weal or woe, in all his thoughts and emotions, and, however much they may be repressed, or disguised, or sublimated, they are really ineradicable.

### Permanent Impressions.

When, therefore, a man has been subjected to emotional shock, or to fatigue sufficiently severe to produce persistent motor, and sensory, and mental disturbances, with such symptoms as insomnia, tremors, depression, loss of memory, it is certain that his nervous system will have received permanent impressions, and not unlikely that the impressions will have permanently impaired its energy and resiliency. A neurasthenic may lose his symptoms, may regain his self-confidence, and may forget that he has been a neurasthenic; but the nervous system has a retentive memory and does not forget, and however fit the man may appear, he may be liable to break down under any considerable competitive strain.

A neurasthenic may remain neurasthenic even after his headaches and his tremors have disappeared; a patient suffering from hysteria may be still hysterical after psycho-therapy has relieved his aphonia; and hundreds of men are discharged from nerve hospitals who seem to be cured, but who are quite unfit to face worry or anxiety. It is well that it should be clearly recognised that nerve injury is not to be estimated by its gross outward symptoms, and that even cases that seem cured may be not only uncured, but incurable. It is well, too, that it should be recognised that the serious feature of neurasthenia is not headache, or tremors, or insomnia, but *neurasthenia*, and that the serious feature of hysteria is not aphonia or muscular contracture, but *hysteria*, and that in some cases it is as impossible to cure completely neurasthenia and hysteria, as to cure completely an amputated leg. Far too much faith has been put in *time*; and effort and money have both been wasted. Now, we must face the fact that a certain small proportion of neurasthenics have been permanently crippled, and, even as pensioners, will not be able to hold their own in the battle of life, and that these chronic cases must be treated as cripples and provided for as cripples.

### How Can We Help?

How can we help the cripples? How can we most economically and effectually make life easy and possible for them, and save them from periodical breakdowns? Ordinary life is competitive and combative, and the incurable neurasthenics are fit for neither competition nor combat: they must, for the term of this natural existence, be put on a non-competitive, non-combatant footing, and it seems to me we can best help them by starting self-supporting communistic colonies where they can live easy co-operative lives. But the colonies must be clearly understood to be not curative colonies, but colonies for incurables.

We hear a great deal nowadays, in socialistic circles, about working for use and not for profit. Whether normal human nature can or should work on non-competitive lines may well be doubted, but here, anyhow, are neurasthenic men who have sacrificed themselves for the present competitive society, and are now unable to compete in the battle of life: why should not society give them an opportunity to experiment with a communistic and co-operative system?

Colonies of various kinds have been started, but, so far, none, I think, has been put on a sound economic footing, because, so far, none has been organised on a communistic basis. The colony I advocate is a self-contained, self-supporting, co-operative colony, producing only what its members require, and using, not selling, its own products. The colony I advocate is not a colony where men are taught trades in order that they may afterwards compete in the open market, but a colony where men may live permanently, making and growing only what they require, and taking no thought of the morrow.

### The Possibilities of Colonies.

This is not the place to enter upon detail, but I suggest that a successful colony for incurable neurasthenics might be founded on the following broad principles.

There are certain things which may be considered necessities for civilised men. Civilised men must, for instance, have boots, food, clothes. Now I propose that there should be just as many men employed in growing food as are necessary to supply all the food they themselves and the other "colonials" require—just as many men employed in making boots as are necessary to keep themselves and the other "colonials" in boots—just as many tailors as are necessary to clothe the community—briefly, just as many land-workers, bootmakers, weavers, tailors, butchers, carpenters, etc., as are needed to supply the whole little community with the necessities of life.

How many acres of land would be required to feed, say, a hundred and fifty neurasthenics, I do not know, nor how many neurasthenic workers would be required to farm each acre. Equally I do not know what would be the right and requisite proportionate representation of

**Co-operative Colonies for Neurasthenics**—(*continued*).

each of the trades; but the matter could be practically worked out—*solvitur ambulando.*

Almost all the necessities of life could be supplied by the work of the "colonials," but certain things, such as coal, raw leather, paper, sugar, salt, tea, tobacco, would have to be purchased. But by a very little excess production on the part of the "colonials" the purchase money could be procured. A few more boots a week than the "colonials" need; a few more eggs a week than the "colonials" can eat; a small excess of production over consumption would suffice to provide money not only for the purchase of such necessities of life as the "colonials" cannot produce, but also to pay for such intellectual and æsthetic provisions as books and newspapers, flowers and concerts.

Suppose, for instance, that a colony consists of a hundred and fifty neurasthenics, and that each does sixpence worth of work daily over and above what is required to supply the daily needs of the community, and that each further pays 5s. a week out of his pension into a common fund. Thereby there would be put annually to the credit of the community a sum of over £3,000, which should, I think, be sufficient not only to pay for such extras as I have already mentioned, but also to cover all administrative expenses and to allow for such accidents as ill-health.

*En passant* I must admit that wives and children would rather complicate the financial and administrative situation, but, since the wives would presumably be healthy women, they should be able to do more than earn their "keep" by various forms of domestic work.

A colony of the kind I have outlined would certainly require very competent administration. A medical director, a lady superintendent, a secretary, and one or two nurses would certainly be necessary, and on their enthusiasm, tact, knowledge of human nature, and power of gaining the confidence and esteem of the "colonials" the success of the colony would almost entirely depend. For the successful management of such a colony personality would be more important even than business ability and cash.

Financially and economically, indeed, there should be little difficulty if land and buildings were provided by philanthropy or by loan on mortgage, and the chief difficulty would be to find a suitable staff and to pick suitable patients, and in the matter of selecting patients the medical director should be given an absolutely free hand. The right medical director must not only be found, but he must be trusted, and he must be given the almost autocratic power of a patriarch under the patriarchal system. He must be the co-ordinating centre and the impelling force of the whole co-operative system, or otherwise disorder, inco-ordination, and insubordination are certain to occur.

Consumptives would not fit so well into such a scheme, since most consumptives suitable for a colony would be curable cases, and would in time recover and return to ordinary life; but, granted certain conditions and adaptations, a place for consumptives might also be found.

